# An overview of Fintech applications to solve the puzzle of health care funding: state-of-the-art in medical crowdfunding

**DOI:** 10.1186/s40854-022-00388-9

**Published:** 2022-09-19

**Authors:** Laura Grassi, Simone Fantaccini

**Affiliations:** School of Management, Politecnico di Milano, Milan, Italy

**Keywords:** Crowdfunding, Health crowdfunding, Pharma, Healthtech, R&D funding, Fintech, Medtech

## Abstract

Crowdfunding is emerging as an alternative form of funding for medical purposes, with capital being raised directly from a broader and more diverse audience of investors. In this paper, we have systematically researched and reviewed the literature on medical crowdfunding to determine how crowdfunding connects with the health care industry. The health care industry has been struggling to develop sustainable research and business models for economic systems and investors alike, especially in pharmaceuticals. The research results have revealed a wealth of evidence concerning the way crowdfunding is applied in real life. Patients and caregivers utilize web platform–based campaigns all over the world to fund their medical expenses, generally on a spot basis, using donation-based or even reward-based schemes, regardless of the health care system archetype (public, private insurance-based or hybrid). Academics have also focused on funding campaigns and the predictors of success (which range from social behaviour and environment to the basic demographics of the campaigners and their diseases) and on social and regulatory concerns, including heightened social inequality and stigma. While equity crowdfunding is disrupting the way many ventures/businesses seek capital in the market, our research indicates that there are no relevant or consistent data on the practice of medical equity crowdfunding in health care, apart from a few anecdotal cases.

## Introduction

The Covid-19 pandemic brought some of the most intricate subtleties in health care to public attention, specifically the timing and riskiness of pharmaceutical research and development (R&D) in its different stages and the methods and levels of funding for R&D. Complexity in R&D is not isolated to vaccines alone but extends to medical care in general and to all high-end pharmaceutical products.

Development of new pharmaceutical products proceeds sequentially, and each step must be satisfied before the compound is approved by health authorities. On average, the entire process takes seven years (Pammolli et al. 2020). Success is rare, with a failure rate of over 96% (Hingorani et al. 2019), caused by economics, lack of efficacy, or safety concerns (Di Masi 2001). Consistently high risks mean that pharmaceutical companies prioritize compound development, given their limited funding^1^ and the need to provide shareholder returns. The social implications of the current schemes are, therefore, of the utmost importance. Among these concerns are a fair price (Balderrama et al. 2020), the varying negotiation skills of governments and insurance companies, accessible health care in every country, regardless of its economic wealth (Proelss et al. 2021), and ensuring that governments and institutions give the highest priority to health research funding (European Commission 2021), as was the case of Covid-19 vaccines.

In this highly complex landscape, crowdfunding − defined as the provision of “financial resources in the form of donations, a future product, service, or some other reward or exchange for shares or debt securities of a company” (Bassani et al. 2019)—is emerging as an alternative mechanism to traditional financing (Cai 2018; Wonglimpiyarat 2017; Kou et al. 2021), which can potentially generate returns (e.g. lending and equity crowdfunding, Martínez-Climent et al. 2018). Crowdfunding in health care, also known as medical crowdfunding (Ren et al. 2020), has already been used in a number of cases. The funds raised have facilitated access to treatment (Kubheka 2020; Snyder 2016), and communities of interest have been built around funded projects (Wilson 2019). However, there are also concerns about exacerbating social inequalities (Berliner and Kenworthy 2017) and the overall effort that is needed (Wilson 2019). Several established crowdfunding platforms regularly host health care-related campaigns: GoFundMe, for example, has reported that one-third of its campaigns are related to medical practice (Allon and Babich 2020).

The purpose of this paper is, therefore, to survey and investigate the literature on crowdfunding in order to describe its relations with the health care industry and identify any gaps in the research, whether empirical or theoretical. With this study, we aim to delve into the foundations of health care industry funding standards, seeking the crowdfunding levers and the effects that crowdfunding has on the innovation and accessibility of medical treatments.

The remainder of this paper is organized as follows. “Theoretical Background” section highlights the extant research and concentrates on the relevant features of the health care industry, exploring innovation in finance, particularly in crowdfunding, and then fills in previous results in literature with additional details. “Methodology” section describes the research methodology. “Results” section gives a first overview of the results, while the emerging themes and trending topics are discussed in “Concept Mapping and Discussion” section. “Conclusions and Implications for Future Research” section contains the conclusions and introduces potential new avenues for research.

## Theoretical background

Health care systems and the pharmaceutical industry undoubtedly have a number of peculiar features. We will introduce the context in this section, with the ensuing limited access to medicines and medical treatments, and present crowdfunding as a Fintech innovation, with particular reference to the financing mechanisms triggered by crowdfunding.

### The health care industry

The odds of successful health care R&D—pharmaceutical in particular—are low, and developmental costs are increasing (Deloitte Centre for Health Solutions 2021; IQVIA Institute for Human Data Science 2021), with manufacturers pursuing a shrinking branded drug market (Drug Channels Institute 2021). This situation creates an environment where financial success is challenging for innovators and potentially results in sub-optimal innovation in the industry. As of today, access to medical treatment is still a key topic in all health care systems in that it is restricted by the suboptimal level of funding. The United Nations’ Sustainable Development Goals agenda considers universal health coverage to be a core right (Kou et al. 2022), defined as all citizens having access to the health care services they need. These services must be of sufficient quality to be effective and to ensure that users are not exposed to any financial burden (Kieny et al. 2017). Public and private health care systems must coexist to ensure this fundamental right, each with its own configuration. Public systems include taxes and social insurance contributions and reimbursements, while private systems are sustained by out-of-pocket expenses and voluntary health care payments (such as elective health insurance, financing from non-profit institutions and enterprise financing) (Immergut and Schneider 2020).

In both systems, bilateral negotiations between pharmaceutical corporations and governments or insurance companies will set the price of each product or treatment. The starting point is either an analysis of willingness to pay or cost-effectiveness studies on opportunity costs that must be understood and interpreted (Siegel et al. 1996). The final price level is also affected by market competition (Cole and Dusetzina 2018; Rosenthal and Graham 2016), promotion—via advertisements, sales representatives and key opinion leaders—to prescribers and end users/patients (Alves et al. 2019), and an integrated mix of basic and applied research (Barigozzi and Jelovac 2020). In the end, an “incredibly complicated and non-transparent environment sets the list price for drugs with very little relation to the true resources used to produce the specific drug” (Nash 2018). Currently, price adjustments are decided at late stages. Ex-post, they need to cover manufacturing and R&D costs, which are constantly climbing due to the complexity of discovering new drugs, and such R&D costs now account for over one billion dollars, including sunk R&D costs for products that failed to pass their clinical trials (Balderrama et al. 2020; Hubbard and Love 2004).

This ex-post pricing mechanism is no longer sustainable. It is not so for the general public, where people have no clear idea of the overall health mechanisms, but suffer from a lack of accessibility and affordability, including having to shoulder cost-sharing arrangements and out-of-pocket expenses (Abbott et al. 2019). It is not so for governments and insurance companies dealing with the health budget, where complex mechanisms and negotiations may hinder more or less confidential agreements or the final price (Henry et al. 2005), and their willingness to follow the entire process with its related uncertainty (Villa et al. 2019). It is not so for society, where access is limited in some countries, mostly those with a lower GDP per capita. Lastly, it is not so for big pharma itself, which, at the current state, relies on IPOs and capital increases to raise money to finance their R&D, while business angels and venture capital firms mostly back smaller companies and their projects; or for pharma company shareholders, where the risks associated to new drug discovery are substantial and so must entail a potential high return (Leadley et al. 2020).

Several ideas have been proposed to stimulate R&D in such a risky environment. Among the most cited are advance-purchase commitments − i.e. agreeing to buy a given amount of a drug when it is developed (Hubbard and Love 2004), incentives or competition for rewards and prizes (Hubbard and Love 2004; Finkelstein and Temin 2008), separation between distribution and marketing (Finkelstein and Temin 2008), patent pools (Bermudez and Hoen 2010; Cox 2012; Childs 2010) and patent buyouts (Kremer 1998). Abbott et al. (2019) put forward several suggestions, ranging from discouraging pay-for-delay agreements to patenting ever-greening, increasing transparency, taxing advertisements that target final consumers for the financing of research, aligning interests on the value chain, and various kinds of revenue-sharing mechanisms above certain thresholds. Moon et al. (2011) analysed tiered prices, i.e. setting the price for drugs systematically lower for emerging countries, arguing that “policies that *de-link* the financing of R&D from the price of medicines merit further attention, since they can reward innovation while exploiting robust competition in production to generate the lowest sustainable prices”.

Fintech^2^ is the natural evolution of the financial services industry enabled by technology (Grassi et al. 2022). It can enhance the process of funding new drug development in smaller pharmaceutical companies that are struggling to find the funds they need to bankroll their research. In larger organizations, it can ensure lower risks along the research process path, which would make its risk-return profile coherent. In other words, crowdfunding, given that it is enabled by the ever-increasing, widespread access to the internet and social media globally, has created new opportunities to access resources, showing terrific potential for both ventures and people in need of assistance (Stevenson et al. 2019).

### Crowdfunding

As an alternative funding source, crowdfunding stands out among the possible Fintech solutions, with a new kind of intermediary (Cai 2018) and where capital is raised directly from a broader and more diverse group of investors. Despite rising attention and the growth of this practice, however, the academic world has struggled to keep up in its crowdfunding literature (Brown et al. 2018; Zhang et al. 2016).

**Fig. 1:**
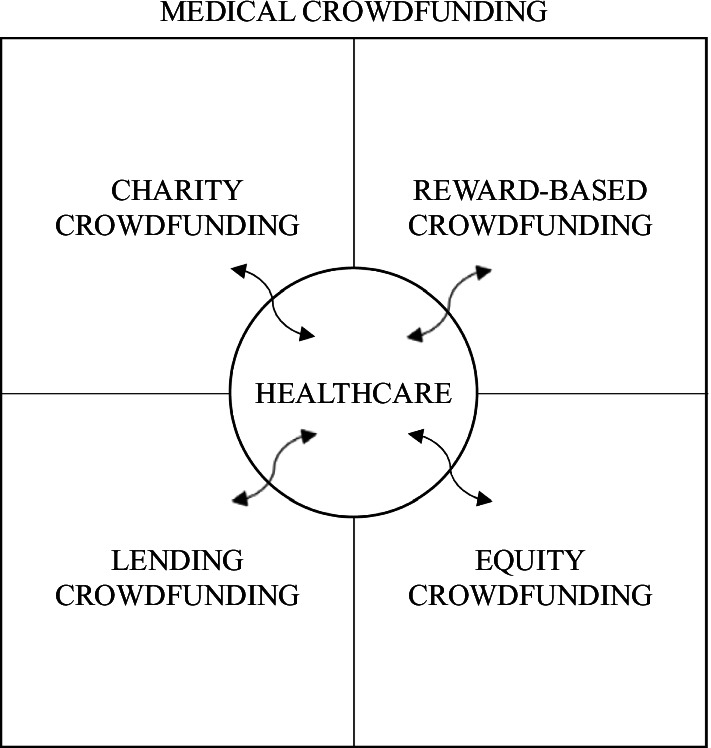
Medical crowdfunding framework

Although there are many different definitions and classifications of crowdfunding, four main archetypes are illustrated in Fig. 1, i.e. charity, reward-based, lending and equity crowdfunding. Charity (or donation-based) crowdfunding is an internet-based non-profit fundraising mechanism for soliciting mostly small monetary contributions from crowd donors to help other people or organizations across the globe in trouble or with dreams (Zhao et al. 2020a). In this specific case, the return for funders can be considered purely social and ethical. Reward-based crowdfunding is another online channel for venture fundraising, where funders receive non-monetary benefits in return for monetary contributions (Shneor and Munim 2019). Especially in the IT industry, the benefit is often early access to the product or prototype. Alongside equity crowdfunding, it is the model most common among start-ups.

Lending crowdfunding (also termed crowd-lending, or peer-to-peer lending) is the practice of liaising between borrowers and lenders through web platforms, by-passing traditional financial institutions (Ziegler and Shneor, 2020); in brief, funders are paid interest for the money they invest, and their capital is returned at maturity or upon the due date. Equity crowdfunding is based on the principle of a digital, online marketplace where entrepreneurs can access a large pool of potential investors who, in return for a stake in the ownership (equity), may fund the ventures (Estrin et al. 2018).

Regardless of the archetype, the key point, as Clauss et al. (2018) observe, is to recognize the drivers (and barriers) for the different stakeholders involved in any crowdfunding campaign, from the people behind the fund-raising campaigns (campaigners) to the potential funders and platform operators. Identification of the predictors of success in crowdfunding has now become the primary focus of research in this field (Short et al. 2017), where the theoretical success factors have been systematically assessed against the campaign outcomes in most areas where this alternative funding method has been utilized.

Scholars have identified the principal predictors of success by investigating the distinguishing features of crowdfunding campaigns both broadly and in detail. They have put together a glossary of definitions identifying terms like crowd and fundraiser (Bagheri et al. 2019; Belleflamme et al. 2014; Ordanini et al. 2011; Ren et al. 2020; Zhang and Chen 2018), building the existing body of evidence primarily on the basis of data from a few large platforms, while smaller markets and emerging entities have often been neglected (Lagazio and Querci 2018; Yu et al. 2017). The lack of depth and breadth has caused the scientific community to question the reliability of these findings when practitioners transfer (or try to transfer) existing knowledge to other domains or platforms (Haasbroek and Ungerer 2020; Stasik and Wilczynska 2018).

In this context, medical crowdfunding is seen as a flourishing opportunity, racketing up reports on a vast array of applications (Mollick 2014; Otero 2015). Despite growing and widespread attention to medical crowdfunding, the evolving economic landscape and the opportunities arising from it, no integrated view of this emerging medium has so far been offered (Lee et al. 2016), to the best of our knowledge.

Hence, a comprehensive review of the relevant literature was undertaken to investigate the literature on medical crowdfunding and so describe its connection with industry-wide health care financing modes. This research also identifies the empirical and theoretical gaps in the research.

## Methodology

We carried out a systematic literature review and clustered the evidence by type to identify significant sub-trends. Lettieri et al. (2009) reported that a systematic literature review is a rational, transparent and reproducible research methodology to analyse extant literature, because the assembling and synthesizing of preceding pragmatic evidence (Delbufalo 2012) can lead to an enhanced descriptive and thematic awareness of the resulting body of knowledge (Sivarajah et al. 2017).

**Fig. 2:**
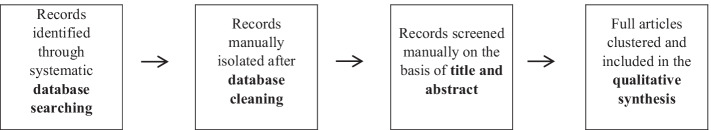
Phases of the systematic literature search and qualitative review

This literature review addressed our objective by setting out the state-of-the-art in crowdfunding applied to the health care industry. The literature was searched systematically to minimize the risk of overlooking potentially relevant contributions (see Fig. 2).

The first phase prepared the ground for the literature review by running a suitably configured database search on Scopus, the largest database of peer-reviewed literature (Nath and Chowdhury 2021). Both crowdfunding and health care domains were included in the search strings, joined by concept mapping and browsing through Thesaurus.com for synonyms, to ensure that the study’s inherent cross-disciplinarity was captured appropriately. As shown in Table 1, the final search query included both “crowdfunding” and “medical”, as well as “health*”, “pharma*”, “drug*”, “medicine*” and “device*”. Based on the SciVal research performance assessment, the results were not limited to a specific timeframe because any focus on more recent years could have caused the loss of significant contributions.

**Table 1:** Literature review database search

Search query:	(crowdfunding AND (medical OR health* OR pharma* OR drug* OR medicine* OR device*))
Database:	Scopus
Keywords in:	title, abstract, keywords
Year restriction:	none
Language restriction:	English
Subject areas:	[Medicine], [Social Sciences], [Engineering], [Business, Management and Accounting], [Biochemistry, Genetics and Molecular Biology], [Economics, Econometrics and Finance], [Pharmacology, Toxicology and Pharmaceutics]
Document and source restriction:	none

## Results

The search identified 196 papers published as of July 14, 2021, filtered by language and subject areas as shown in Table 1. The relevant items were reduced to exclude duplicates (1 paper), records that had slipped through the filters (1 Spanish article) and contributions that were not otherwise identifiable (missing authors, sources, or DOI, 19 cases).

The inclusion criteria were verified to ensure that, when applied to the rough database obtained after the first cleaning; they would extrapolate data that were as relevant and specific as possible. To assess each item’s eligibility, the titles and abstracts were reviewed according to two main criteria. Firstly, we considered only explicit health care references, excluding any kind of non-medical application (38 non-eligible papers, e.g. references to financial or economic health). Secondly, we selected studies that investigated the specific field of crowdfunding (22 non-eligible papers, e.g. crowdsourcing as a general process whereby a firm outsources pieces of a given work and leverages on hard skills found within the general public, the crowd). No further screening between the crowdfunding archetypes was necessary, given our objective. Most of the final 115 papers selected were articles (81); there were also a few notes (9), reviews (8), letters (6), short surveys (5), editorials (3), conference papers (2) and one book chapter.

The analysis of the final sample showed that the earliest publication year was 2012, which is consistent with the relatively recent field of studies on crowdfunding in health care. The concentration in publications is unbalanced towards the more recent years, with 57% of the items published from 2019 to 2021, highlighting the vitality of the topic under discussion. Of the papers selected, 62 focused mainly on the global situation or had no connection to specific geographical areas, and 53 referred to local specificities, with 27 papers covering cases in the USA, 10 in Canada, 4 in Europe and 3 in the UK; in the Asian area, 7 papers covered cases in China and 2 in Indonesia.

Twenty-three publications were linked to economic and social science outlets (“Business Management and Accounting” and “Social Sciences”) and 9 to engineering fields and multidisciplinary journals, but for the vast majority (83 items), the outlets for publication were health care-related journals (“Medicine; Pharmacology, Toxicology and Pharmaceutics; Biochemistry, Genetics and Molecular Biology”) (Table 2). This is consistent with the current scope of the research papers analysed in this review, where the focus was mainly on charity crowdfunding to support out-of-pocket medical expenses incurred by individuals with rare or severe diseases (81 charity crowdfunding papers overall, 58 in health care journals). The remaining 34 items refer to crowdfunding in general. Some of the items made no mention of archetypes or any other classification (21 overall, 16 in health care journals); others discussed all four archetypes (8 overall, 5 in health care journals), or focused on a specific model, where 3 covered lending and equity crowdfunding (of which 2 were in health care journals) and 2 covered reward-based crowdfunding.

**Table 2:** Crowdfunding archetypes and research subject areas

	Medicine; Pharmacology, Toxicology and Pharmaceutics; Biochemistry, Genetics and Molecular Biology	Social sciences	Business, Management and Accounting	Multidisciplinary	Engineering
Charity crowdfunding	58	14	3	4	2
Reward-based crowdfunding	2	–	–	–	–
Lending crowdfunding	2	–	–	1	–
Equity crowdfunding		–	–		–
All the archetypes	5	1	1	–	1
No reference to any classification	16	3	1	1	–
TOTAL	83	18	5	6	3

We began by analysing all the items extrapolated from the systematic search through the lens of the major crowdfunding archetypes. Interestingly, this analysis revealed that there is much discussion on charity- and donation-based models, which possibly provide limited support to the funding of health care R&D, given the small amounts raised compared to the overall picture. Furthermore, there is no undisputed evidence on the practice of lending and equity crowdfunding in health care. To expand our understanding of crowdfunding-based mechanisms in health care, we went back over the final sample and re-clustered all the records manually, using an inductive approach based on the research domain, the main objective and the primary findings of each contribution. These findings are presented in “Methodology” section.

## Concept Mapping and Discussion

In setting out our results, we opted for a thematic discussion (Tranfield et al. 2003) to identify the research themes, contribute to the understanding of the research field detected through our analysis, and also to explore future directions of research (Xu et al. 2019). The detailed analysis of the studies under review prompted us to divide the papers into four groups according to the themes that emerged in the selected body of knowledge (see Table 3). The four groups are a) crowdfunding as an alternative instrument to access medical treatment (17 contributions), b) support to medical research and development (32), c) performance reviews of platforms and funding campaigns (26) and d) policy and ethics concerns and considerations in the field of medical crowdfunding (40). The resulting framework forms the basis for the presentation and discussion of our findings.

**Table 3:** Descriptive information for studies included in the systematic review

CROWDFUNDING IN HEALTH CARE, 115 results	
*Alternative access and pitfalls in health care systems, 17 results*	
Cohen et al. (2019)*; *Coutrot et al. (2020)*; *Di Carlo et al. (2020)*; *Ho et al. (2019)*; *Imanulrachman et al. (2019)*; *Kenworthy (2019)*; *Kimseylove et al. (2020)*; *Lublóy (2020)*; *Lukk et al. (2018)*; *Palad and Snyder (2019)*; *Rajwa et al. (2020)*; *Renwick and Mossialos (2017)*; *Saleh et al. (2021)*; *Sisler (2012)*; *Snyder et al. (2020a)*; *Snyder et al. (2020c)*; *The Lancet Oncology (2017)	
*Support to medical research and development, 32 results*	
Alternative sourcing	23 results
Afshinnekoo et al. (2016)*; *Byrnes et al. (2014)*; *Cameron et al. (2013)*; *Chetlen et al. (2018)*; *Dragojlovic and Lynd (2016)*; *Gallerani et al. (2019)*; *Hidayat et al. (2020)*; *Kamajian (2015)*; *Koole et al. (2018)*; *Krittanawong et al. (2018)*; *Makris (2015)*; *Otero (2015)*; *Özdemir et al. (2015)*; *Ray and Özdemir (2016)*; *Riccardi et al. (2017)*; *Schucht et al. (2020)*; *Schuhmacher and Kuss (2020)*; *Sharma et al. (2015)*; *Sifferlin (2015)*; *Siva (2014)*; *Smith and Merchant (2015)*; *Weigmann (2013)*; *Wiebe and FitzGerald (2017)	
Neglected diseases and orphan drugs	9 results
Del Savio (2017)*; *Dragojlovic and Lynd (2014)*; *Fumagalli and Gouw (2015)*; *Hahn (2015)*; *Isakov et al. (2015)*; *Loucks (2013)*; *Rajput et al. (2015)*; *Tóth et al. (2021)*; *Verbaanderd et al. (2021)	
*Performance review of platforms and funding campaigns, 26 results*	
Predictors of successful funding campaigns	24 results
Aleksina et al. (2019)*; *Ba et al. (2021)*; *Berliner and Kenworthy (2017)*; *Durand et al. (2018a)*; *Durand et al. (2018b)*; *Fong et al. (2020)*; *Fuguo et al. (2021)*; *Holmes et al. (2019)*; *Huang et al. (2021)*; *Loeb et al. (2018)*; *Park (2012)*; *Peng et al. (2021)*; *Pol et al. (2019)*; *Proelss et al. (2021)*; *Ren et al. (2020)*; *Saleh et al. (2020)*; *Saxton and Wang (2014)*; *Solotke et al. (2020)*; *Thomas et al. (2021)*; *Thompson et al. (2015)*; *Vassell et al. (2020)*; *Xing et al. (2021)*; *Xu and Wang (2020)*; *Zhao et al. (2020b)	
Crowdfunding platforms	2 results
Bassani et al. (2019)*; *Besancenot and Vranceanu (2019)	
*Policies and ethics, 40 results*	
Gonzales et al. (2018)*; *Snyder and Crooks (2020)*; *Snyder et al. (2016)*; *Snyder et al. (2021)	
Non-approved treatments	13 results
Iqbal and Collins (2020)*; *Murdoch et al. (2019)*; *Smith (2015)*; *Snyder and Caulfield (2019)*; *Snyder and Cohen (2019)*; *Snyder and Turner (2019)*; *Snyder and Turner (2018)*; *Snyder and Turner (2021)*; *Snyder et al. (2020b)*; *Song et al. (2020)*; *Tanner et al. (2019)*; *Vox et al. (2018)*; *Zenone et al. (2020)	
Regulatory concerns	9 results
Dressler and Kelly (2018)*; *Jin (2019)*; *Kubheka (2020)*; *Mercer (2019)*; *Moore (2019)*; *Ross (2020)*; *Young and Scheinberg (2017)*; *Zenone and Snyder (2019)*; *Zonia (2016)	
Social inequalities	14 results
Barcelos (2019)*; *Barcelos (2020)*; *Barcelos and Budge (2019)*; *Burtch and Chan (2019)*; *Igra et al. (2021)*; *Kenworthy (2021)*; *Lee and Lehdonvirta (2020)*; *Paulus and Roberts (2018)*; *Silver et al. (2020)*; *Snyder (2016)*; *Snyder et al. (2017a)*; *Snyder et al. (2017b)*; *Van Duynhoven et al. (2019)*; *Zenone and Snyder (2020)	

### Alternative access and pitfalls in health care systems

Today, nearly 2 billion people have no access to basic medicines, with the ensuing fallout in preventable misery and suffering (World Health Organization 2017). While the world is struggling with access to medical care, there are serious misgivings that the current funding and business models (especially in pharmaceuticals) will break health system budgets as worldwide spending outpaces both overall health expenditure and economic growth (Moon 2017). It is, however, worth pointing out that what has recently become headline news in high-income countries has long been a concern everywhere else: unaffordable medicines and inadequate innovation have become global issues, and business as usual is no longer an option. Considerations of accessible treatment are one burning theme that emerged from the studies into current crowdfunding in health care.

Health is a fundamental human right, and its four core principles are availability, accessibility, quality and equality (World Health Organization WHO 1946). However, access to health care is most times prevented by the patients’ financial circumstances. Kimseylove et al. (2020) demonstrated that there are significantly more patients setting up online campaigns to fund transgender medical services in areas of the United States where these practices do not fall within the health coverage programmes for low-income resident procedures.

Costs remain one of the major constraints for these patients, and crowdfunding could encourage and facilitate “right to try” practices (the American “Right-to-Try” experimental drugs act of 2018), although the evidence would suggest that there is no practical benefit compared to the more regulated U.S. Food and Drug Administration (FDA) expanded access programmes (Snyder et al. 2020a). Imanulrachman et al. (2019) studied a number of such campaigns in Indonesia and concluded similarly that crowdfunding is a viable and fair alternative for accessing health care services, in the respect of all WHO guiding regulations. A point to highlight is that the local contexts in the two studies show that accessibility challenges affect patients all over the world, and not just those in low-income countries.

The advantages of better access will not apply just to the patients, but also to health care systems in general. Renwick and Mossialos (2017) argued that crowdfunding may bring many economic benefits to health care, especially apropos of deferred and underserved medical issues. Many of the benefits of crowdfunding − improving access to funding and social engagement, to name a few − are common to all cross-sector applications; however, as previously outlined, health care usage should be handled more carefully, because alternative paths of access are potentially disruptive and could upset the broader priority settings in public health.

Crowdfunding campaigners are usually motivated by gaps in the wider social system, like travelling costs related to medical care or unpaid time off work (Snyder et al. 2020c), and this applies even where the health care system is universal and publicly funded, in Canada, for example. Similarly, other authors have argued that the spread of health care crowdfunding is nothing but a shortcoming in Canada’s provision of state welfare (Lukk et al. 2018). Comparable investigations were conducted all over the world and across all health care systems, including in the United Kingdom (Coutrot et al. 2020), Germany (Lublóy 2020) and the United States (Sisler 2012). The latter, in that it is a typical private system where people pay directly (out-of-pocket) and the lucky ones have insurance, deserves a special mention, because there is an underlying trend to link health care crowdfunding to the lack or breadth of health insurance coverage. In several circumstances, a crowdfunding campaign is not even the medium to obtain the treatment or service but the only way to pay for everyday costs and avoid the yoke of medical-related bankruptcy.

The gaps in health care systems that emerged from studying crowdfunding applied to health care have been investigated in the various fields of medicine and across geographic areas. Oncology stands out in particular because technological innovation and epidemiological factors have all contributed to a surge in costs and, hence, the financial burden on patients (Cohen et al. 2019). Chimeric antigen receptor T cell (CAR-T) therapies, which is the new frontier in cancer treatment, have also been analysed in several studies, with evidence suggesting that patients may have to face unforeseen indirect costs associated with their treatment and should consequently be advised on all the potential means to handle these costs—even through unconventional resources like crowdfunding (Ho et al. 2019). Similar examples can be found in other therapeutic areas, from urology (Di Carlo et al. 2020) to drug abuse and addiction-related services (Palad and Snyder 2019).

The extraordinary circumstances of the Covid-19 pandemic have added to the woes in economic and health care systems, hampering access to medical care. Reports indicate that the online crowdfunding response has been remarkable, with an exponential rise in patients relying on web campaigns to fund their costs − mostly related to services or protective equipment (Rajwa et al. 2020). Governments and policy-makers could peruse this practice, and their thoughtful input could be the pulse-check tool to spot underserved needs and define social distress more accurately, especially in the face of a global health threat (Saleh et al. 2021).

Different views emerge on medical crowdfunding, seen as a complex innovation—but not simply and purely a good one—that is reshaping systems, influencing disparities, even shifting political norms (Kenworthy 2019), or as a practice (just like charity) meant to be a last resort, or even signalling the failure of health care systems, especially those that are universal and publicly funded (The Lancet Oncology 2017).

### Support to medical research and development

A second trending theme that has emerged from our review is crowdfunding applied to medical research and development. In general, crowdfunding functions as an alternative or complementary source of funding for scientists and small ventures. In particular, it is a novel way to give financial backing to the development of orphan drugs and the treatment of underserved diseases when private or public spending is dwindling (Sifferlin 2015; Siva 2014; Weigmann 2013). No papers in this area refer specifically to equity or lending, and most studies present an overarching perspective on crowdfunding or allude to donation-based (charity) crowdfunding.

#### Alternative sourcing

In recent years, health care researchers have relied increasingly on crowdfunding to support their work, leveraging on the crowd’s personal reasons for seeing these initiatives take off and, in a few cases, a general appetite for some type of financial and monetary reward. Examples and preliminary explorations are described in the niche areas of genomics, bioinformatics, microbiome and meta-genomic research, up to broader domains in medical practice, such as infectious and cardiovascular diseases or the field of neuroscience (Afshinnekoo et al. 2016; Cameron et al. 2013; Riccardi et al. 2017; Krittanawong et al. 2018; Schucht 2020). When studying crowdfunding principles applied to medical research there is a common sentiment that could drive innovation in health care and public involvement in biomedical research (Kamajian 2015; Makris 2015).

Some research focuses on the predictors of success in this specific area, similarly to the case of single campaigns or platforms. Dragojlovic and Lynd (2016) studied drug development campaigns in North America to determine the preferences of prospective donors. Although there is a predilection among donors for non-profit research organizations, the authors’ findings suggest that the crowd is not averse to donating to benefit corporations. Crowdfunding can support early-stage biotech ventures and seed funding, in particular when the market potential of the assets at stake has yet to be established (Dragojlovic and Lynd 2016). Evidence from Chetlen et al. (2018) and Sharma et al. (2015) confirms this hypothesis and demonstrates that these limited funds are often used to trigger and finance small initiatives. They serve as a bridge and a complement to larger grants or funding, and support early-stage clinical trials. While this approach can assist small new ventures, it can also be relevant for large, established pharmaceutical companies by mobilizing the general public through crowdfunding to prioritize and focus on what the system really values (Schuhmacher and Kuss 2020).

While crowdfunding campaigns cannot yet replace the more traditional funding mechanisms for medical research, the evidence indicates that these small sums of money can act as a starting point and ensure the survival of early-stage start-ups and scientific funding in general (Gallerani et al. 2019). Campaigners are required to bring to the cause a certain ability in the matters of social media engagement, beyond the necessary technical knowledge of the medical science in question and the ability to translate it into the language of the crowd (Otero 2015). Building an audience, actively engaging with it, nurturing and expanding it, is key to success, like in any other crowdfunding application (Byrnes et al. 2014). Medical scientists, like all other campaigners, have to push their visibility and consistently increase their social media presence, putting their best efforts into identifying and engaging with key stakeholders in the relevant community, and look beyond their specific interests if they are to gain tangible financial support (Smith and Merchant 2015). This approach seems to apply broadly, regardless of the disease and research area. Successful cases in the domain of heart disease are reported to be critically linked to the campaigners’ ability to establish connections with professional organizations (patient advisory groups) and, also, to their ability of delivering the message through a simple and straightforward narrative (Koole et al. 2018).

The current body of evidence provides an interesting perspective on medical crowdfunding in terms of social participation and the service it can provide to the community. Medical crowdfunding can be a way the general public can directly address major health problems in their communities. In order to achieve this vision of social empowerment, current funding models should be reshaped, with a concerted effort to bring angel investors and common citizens together and pool their resources for the good of public health and innovation, whilst, equally, contemplating a favourable return on their investment (Özdemir et al. 2015).

The Covid-19 pandemic has aggravated the need for a different approach to health care funding, and there are practical examples of how crowdfunding can help to fast-track the development of medical assets under extraordinary circumstances by leveraging community spirit, social entrepreneurship and the crowd’s collaboration (Hidayat et al. 2020). On a scale larger than single-case applications, the socialization of health care funding and the empowerment of financial communities have the potential of massively reshaping the direction of research, beyond patient centricity and partnerships in trial design and patient enrolment, with all the ethical and integrity questions brought up by this chain of events (Wiebe and FitzGerald 2017). Ray and Özdemir (2016) seem to share the same view when they claim that crowdfunding has the potential to fill current gaps in health care systems through large-scale social engagement, although in some countries like China or India, this kind of financial contribution is still very uncommon despite their vast populations.

#### Neglected diseases and orphan drugs

Orphan drugs are pharmaceutical agents designed to prevent, diagnose and treat diseases (U.S. Food and Drug Administration 2018) which are considered “rare”, with a low prevalence of about 1 in 1,500 people (U.S. Congress 2002), despite affecting more than 300 million people globally, or which, more generally, are underserved because of missing resources or support to research and development, thus classifying them as “neglected”.

Crowdfunding is considered an effective alternative to fund and boost medical research in orphan drugs, an area currently facing significant limitations in resources (Rajput et al. 2015).

Despite the many incentives provided by the FDA, including longer patent registration times and tax breaks, drug development is an expensive business, and orphan drugs are no exception. Hence, extra funding to early-stage biotech companies is key to success (Loucks 2013), and medical crowdfunding could become a welcome surrogate for financing innovation (Dragojlovic and Lynd 2014).

As further discussed in the section on policies and ethics, it is the opinion of some that the massive usage of crowdfunding methods in the medical research domain may lead to system-wide short cuts in existing expert-based scientific evaluation processes, like choosing to prioritize resources on the basis of the disease burden. Others (del Savio 2017) strongly believe that this practice has the potential to bring new life to this industry, especially in areas affected by persistent standard funding failures, including neglected diseases in general, or specific, underfunded and currently incurable diseases like Alzheimer’s and Parkinson’s (Tóth et al. 2021). A number of different and specific cases have been reported, from rare genetic conditions to pre-eclampsia (Fumagalli and Gouw 2015; Hahn 2015; Isakov et al. 2015). An alternative funding perspective and an altered private–public mix in the financing of R&D may bring new life to a number of compounds in different disease areas (e.g. different types of cancer, Parkinson’s disease), which are currently deprioritized because of their poor commercial prospects (Verbaanderd et al. 2021).

### Performance review of platforms and funding campaigns

The third dominant theme emerging from the review is performance, namely a focus on funding campaigns and the predictors of a successful funding campaign − from social behaviour and environment to the campaigners’ basic demographics and their diseases − and the best platforms to use for medical-related campaigns from one country to another. We have centred our discussion on the two main trending areas, i.e. the predictors of success across different geographical and disease areas and the platforms used (plus any complementary technical feature) and their correlation to campaign performance. Overall, our findings in Sect. 5.3 are in line with other theoretical and empirical contributions in the general field of crowdfunding, where the attention of scholars and practitioners has been primarily driven by key success factors for campaigners and the platforms’ performance (Clauss et al. 2018; Short et al. 2017).

#### Predictors of successful funding campaigns

The predictors of success in medical crowdfunding campaigns have been studied from many angles, in various countries and under different circumstances. A number of factors have been shown to affect the output of a single initiative dramatically. These range from interpersonal relationships (social media presence and contact networks), reciprocity in helping, attitude towards donating (target audience), perceived control of behaviour, perceived trust, project information and the patients’ characteristics (Huang et al. 2021; Xing et al. 2021). In China, medical information (low mortality rate, high frequency) and epidemiological details about the campaigners and clinical cases were found to be among the most reliable determinants of success, and the same holds for some demographic and social attributes, like age and location (Ba et al. 2021). The association between demographics linked to the project initiators and epidemiological characteristics linked to the disease, on one side, and funding success, on the other, was also assessed thoroughly in the United States, Canada and the United Kingdom (Saleh et al. 2020).

Although the reasons behind each individual campaign look quite different in different countries, and social disparities have emerged from the study, medical campaigns raised more funds in the United States in general, with black people, women and routine care being usually less successful. It is worth noting that campaigns for inaccessible and experimental care, more common in cancer treatment, have raised more than for routine care (Saleh et al. 2020). Machine learning was brought into play to test these individual factors on the largest scale and provide more clarity on their actual impact on fundraising performance and on the potential patterns and clusters, and to predict the amounts that will be raised (Peng et al. 2021).

The determinants of the success of medical crowdfunding campaigns in specific disease areas were also investigated. Certain conditions seem to produce a better performance than others, even within the same disease area. This is the case for organ transplantation, where liver transplants yielded half the campaign target, on average, versus kidney transplants, which reached a mere 11.5% (Pol et al. 2019). An analogous situation can be observed for cancer treatments and complementary costs. These medical crowdfunding campaigns are most often triggered and managed by relatives or friends of the affected, with breast cancer campaigns raising more funds than those for prostate cancer (Loeb et al. 2018). Malignancies in general tend to attract donor attention, as evidence from thyroid surgery-related crowdfunding suggest, with thyroid cancer campaigns raise more than other diseases in the same area (Fong et al. 2020).

Narratives about how the initiators and promoters describe the illness and financial need play a key role in determining the success of a medical crowdfunding campaign. Different authors have highlighted the importance of a compelling and accurate narrative, starting from the project title, mastery of medical jargon, accurate but not overly technical wording, third-person storytelling and an appropriate story length, a positive emotional sentiment in the narrative, and setting a higher fund target (Durand et al. 2018b). Beyond the medical technicalities, a successful campaign uses very personal and emotional wording, centred on the loss of quality of life, lack of support and care, and what is sought and hoped for (Berliner and Kenworthy 2017; Fuguo et al. 2021; Vassell et al. 2020; Xu and Wang 2020).

On average, medical crowdfunding campaigns perform better when the subject is an infant girl, or children, in general (Ren et al. 2020) and the words used are accurate without being technical. Another feature that plays in favour of a better outcome is to use images of the patient, especially when diagnosed with more severe diseases. The most successful campaigns are usually launched around holiday time, with a peak during Christian religious holidays (Proelss et al. 2021). Similar trends apply in the sphere of some rare diseases, where Vassell et al. (2020) systematically studied medical crowdfunding campaigns in support of the diagnosis and treatment of Lyme disease, looking for common themes in these narratives. Research in the area of orthopaedics and oncology—kidney cancer in particular—produced similar results, confirming that the allocation of resources (donations) is disproportionally biased towards some diseases, locations and people who know how to master social media, leveraging on their networks to produce a compelling project narrative (Durand et al. 2018a; Thomas et al. 2021). Food relief campaigns in the United States connected to Covid-19 were also investigated, highlighting once again that a successful output is the result of an exquisite combination of wording, imagery and presence on social media (Zhao et al. 2020b).

The internet is the natural environment of every medical crowdfunding campaign. By definition, it is the place where all crowdfunding campaigns live and prosper, and many authors describe how the ability of campaigners to build and leverage on the social media effect is key to ultimate success (Aleksina et al. 2019), joined by proficiency in media language (Berliner and Kenworthy 2017; Fuguo et al. 2021). Online donors behave differently. They tend to follow the media stream more than anything else and have a preference for certain categories rather than others (health care in particular), and they give small donations (Saxton and Wang 2014). By exploiting social media contents, a strong social media network will play an important role in all medical crowdfunding campaigns (Park 2012) in general, and in the area of oncology and haematology in particular (Thompson et al. 2015). Regardless of the type of disease or its location and although media attention still emerged as a significant predictor of success, certain items seem to be less attractive than others, with pharmacy-related products (and anything relating to patient medication and medication management) appearing to have a low success rate (Holmes et al. 2019).

If a certain condition is better socialized, the success of medical crowdfunding is more likely, whether media attention is focused on a disease or on the associated financial burden that people may face, and these same aspects can affect a campaign negatively and put it on the path to failure. Evidence from a recent study shows that the success rate for crowdfunding campaigns to support abortion services is extremely low in the United States, regardless of the abortion policy of the state in question (Solotke et al. 2020). Despite the fact that a third-person narrative raised significantly more money in general, especially when the abortion was triggered by the diagnosis of high maternal or foetal risk of an ensuing negative outcome, patients and campaigners should be aware of the media effect that could amplify the social stigma around some conditions.

#### Crowdfunding platforms

Research shows a direct correlation between the spread of medical crowdfunding platforms and health care system archetypes, with authors describing a real substitution effect when coverage is poor (Bassani et al. 2019). Most studies concentrated on a single platform, or a few at most, and in one study, the authors took a comprehensive approach to investigating the platforms and environmental factors that influence them (Bassani et al. 2019). The selection of a platform is directly related to the possibility of running a successful campaign, and evidence suggests that investment-based platforms (e.g. equity crowdfunding) and those dedicated to health care matters are less likely to be successful when used in medical crowdfunding campaigns (Bassani et al. 2019).

Furthermore, considering all-or-nothing crowdfunding platforms, where campaigners are funded only if a pre-defined target is reached, Besancenot and Vranceanu (2019) demonstrated that these tools could encourage inefficient crowdfunded financing, where the emotional component is generally the main driver for donating, regardless of the financial feasibility (and efficiency) of the project promoted.

Our study suggests that the evidence on platform performance and funding campaigns in health care has been collected around only a few platforms, possibly in line with what has been reported for general crowdfunding (Yu et al. 2017), because only a small number of platforms can supply enough data for meaningful quantitative research. In fact, because of the ease of access to huge volumes of data, Kickstarter and other top-trending platforms were the only ones really suitable to study crowdfunding (Yu et al. 2017). Various authors claim that this small pool of source material has limited their research (Chan et al. 2018; Colombo et al. 2015; Cox and Nguyen 2018; Wang et al. 2018), meaning that their findings are only partially applicable to other platforms, regions, or markets or not at all (Stasik and Wilczynska 2018). Based on our findings, the same limitation applies to medical crowdfunding.

### Policies and ethics

Regulatory and ethical concerns are the most common topics when carrying out a systematic review of the literature on crowdfunding in health care (Snyder et al. 2016). Markets and services are evolving rapidly and regulating such an environment requires policy-makers to have adequate technological instruments at their fingertips, hence this discipline is today (and tomorrow) key to the very survival of the broader ecosystem. While some authors question the privacy of patients and campaigners (Gonzales et al. 2018; Snyder and Crooks 2020), most seem to be concerned with the use of medical crowdfunding to support non-approved or alternative treatments, the lack of a regulatory framework in which to operate, and the widening of social disparity as the result of the unsupervised practice of crowdfunding in health care (especially donation-based, single-patient applications). Interestingly, Covid-19 has been playing its own role, raising concerns about the medical crowdfunding medium and highlighting how its misuse can be a source of misinformation (Snyder et al. 2021), which can pose a threat to global public health efforts and be a waste of resources.

Considering the different archetypes of crowdfunding, equity crowdfunding is an outlier when looked at from an investor’s perspective. Equity crowdfunding implies investment decisions with the prospect of a potential return on investment, meaning higher risk levels compared to reward-based crowdfunding, where, as mentioned, funders get material or immaterial rewards for their financial support or a refund if the funding campaign does not reach its goal. Also, equity crowdfunding is largely associated with information asymmetries in the evaluation of new ventures because most funders are retail non-professional investors (Mochkabadi and Volkmann 2020), with limited knowledge of funding mechanisms, shareholder duties and the ensuing risks. Therefore, in this world of rapidly evolving financial markets and services, policy-makers who regulate this environment must have the right technological tools at their disposal. The implication is that appropriate policy-making is critical to the survival of the broader ecosystem, both now and in the future.

#### Non-approved treatments

A large body of data on medical crowdfunding is being used to request financial support for unlicensed drugs or alternative therapies in general, especially in North America and especially for cancer treatment (Iqbal and Collins 2020). These campaigns are often supported by and through the news media. In a recent study by Murdoch et al. (2019), roughly 20% of the articles from a combined sample of newspapers from the United States and Canada referred to treatments that are either unproven or lack regulatory approval.

The findings suggest that campaigners often rely on crowdfunding to access alternative or complementary treatment alongside licensed medicines, as substitutes or because licensed treatments are not available (Snyder and Caulfield 2019). Among other risks, this practice could determine intrinsic inefficiency in allocating resources, especially when the driver for campaign success is a misleading narrative (Snyder et al. 2020b). A practical example is the crowdfunding of cannabidiol for cancer-related care, where most campaigns are backed by anecdotal evidence and misinformation is widespread (Zenone et al. 2020). Vox et al. (2018) assessed a broader set of treatments and, again, the study demonstrated that medical crowdfunding is widely used to finance unlicensed or ineffective—and sometimes potentially dangerous—treatments within a range of disease areas.

Although these campaigns are usually less successful than medical crowdfunding for approved treatment, the way the funds are spent is far from virtuous (Vox et al. 2018). A case in point is crowdfunding for stem cell treatment, a therapy employed in a number of different diseases, with campaigns being relatively successful despite this practice being just a fraction of the total number of crowdfunding campaigns. Nevertheless, most treatments are unproven and misrepresented, and donations seem to be triggered by misleading narratives often intended to tug at the heartstrings and tap into a perceived scientific value (Snyder and Turner 2018, 2021; Tanner et al. 2019).

Several authors have suggested different ways of mitigating and regulating the practice, starting with stem cell treatment crowdfunding, where Snyder and Turner (2019) backed the idea of targeted patient education initiatives and policies to raise awareness and limit misuse. Education is key, not just to educate patients but, more broadly, for all the stakeholders, including the doctors, who should learn about complementary therapies (Song et al. 2020) and the practice of crowdfunding and alternative medical funding, in general. Other authors suggest that the crowdfunding platform should shoulder the burden of implementing stronger regulations and so avoid spreading misinformation and funding unproven medical therapies (Snyder and Cohen 2019). The perspective put forward by Smith (2015) is extremely interesting, where he advocates for the health authorities’ early intervention (e.g. FDA) in giving guidance to small companies seeking alternative funding.

#### Regulatory concerns

Given the right circumstances, medical crowdfunding can be a formidable instrument for serving the health care ecosystem and all interested stakeholders. It will not be easy to curtail the risks and embed a fair regulatory framework into crowdfunding. There are too many forces at stake, including ambiguity in laws and policies and a lack of control, reporting and awareness (Young and Scheinberg 2017). At its worst, absentee governance could leave room for deception and crowdfunding fraud. Today, there is little or no evidence of this kind of regulatory framework across the geographical and social spectrum and, although platforms have policies to protect both donors and campaigners, some argue that this is not enough (Jin 2019; Zenone and Snyder 2019). It has been claimed that “virtuous donors” (i.e. the crowd policing itself and being self-rigorous) could be a deterrent against crooked campaigns in health care and mitigate the associated ethical risks (Moore 2019). Another crucial point to consider is how medical crowdfunding apparently influences policy-making at many levels, from safeguarding patients and donors to mitigating conflicts of interest in federally or nationally funded medical research (Zonia 2016).

The recent and unfortunate case of an infant boy in the United Kingdom affected by a rare genetic disorder triggered animated discussion within the medical community and captured public opinion (Ross 2020). Beyond the ethical concerns relating purely to medical practice, the authors observed that these situations can be the driver to reallocate social resources (Dressler and Kelly 2018). More importantly, if applied on a large scale, especially in publicly funded systems, medical crowdfunding could, in fact “commodify” health care. Introducing properly regulated market norms where there are none is not necessarily bad, especially in response to structural issues (Mercer 2019). It is also true that the current market is widely influenced by unfairly distributed forces, such as social media savviness, network engagement and the ability to create compelling narratives (Kubheka 2020), despite the fact that all of this should have no say in how essential medical services and goods are accessed.

#### Social inequalities

Medical crowdfunding campaigns are more successful for those who are wealthier, have better access to interpersonal wealth and social media, and are more able to appeal to the crowd of donors and outpace the competition (Lee and Lehdonvirta 2020; Silver et al. 2020). Related studies carried out in the United States have exposed the way funds proffered by the crowd are not distributed universally or based on need, rewarding the more privileged and widening socioeconomic gaps in access. The same conclusion is supported by a recent study of Canadian cancer-related medical campaigns, where the authors demonstrated that crowdfunding usage is disproportionately linked to those with higher income and education (Van Duynhoven et al. 2019). Medical crowdfunding as an amplifier of social inequality is a hot topic that is well-represented in the existing literature. There is mounting concern among the authors about how the practice as it is today—an alternative or complementary path to access medical care—may be the cause of system-wide injustices rather than a solution (Snyder 2016; Paulus and Roberts 2018).

Another topic which has emerged is that social stigma influences campaign performance. The literature has been thorough and comprehensive in describing the outcomes when campaigners represent more vulnerable and socially stigmatized groups, such as transgender people or women requesting an abortion (Kimseylove et al. 2020; Solotke et al. 2020). The evidence suggests that these campaigns are regularly unable to reach their goals, achieving roughly 25% of their target in a selection of transgender medical crowdfunding cases (Barcelos 2019), while leveraging on social networks in this way could instead aggravate the (social) burden on these groups (Barcelos 2020; Barcelos and Budge 2019; Zenone and Snyder 2020).

Technological and social gaps could also create an unfair market and fuel inequalities.

If crowdfunding can truly reduce personal medical-related bankruptcy in non-publicly funded health care systems, like in the United States, social stigma may be more extensive than it seems at first glance and embrace all “digitally divided” disadvantaged groups (Burtch and Chan 2019). These people are not only affected by their medical condition and inability to access the most basic health care but also by system-wide disparities in the usage of crowdfunding and its outcomes. The end result is only marginally influenced by elements under the campaigners’ control (e.g. the patient narrative or complementary treatment), while the crowdfunding economy and socio-technological factors reinforce inequality in the spotting and addressing of medical care gaps (Kenworthy 2021). Covid-19 acted as a catalyst, in this case compounding inequalities in society by favouring privileged groups of individuals with better access to the digital market (Igra et al. 2021).

Crowdfunding health care has the potential to disrupt the way we access medical care, yet there are ups and downs and good and bad elements. If not properly controlled and regulated, the existing gaps may widen and, paradoxically, the pitfalls and shortages in both the economic and the health care systems may be masked (Snyder et al. 2017a, 2017b). It is, however, important to consider that current evidence refers specifically to donation-based (mostly single-case) applications or crowdfunding in general, while there is still a huge void in areas specifically linked to medical equity (or lending) crowdfunding.

## Conclusions and Implications for Future Research

### Contributions

The results of this paper show that crowdfunding is a trending health care topic. Across the world, patients and caregivers are putting their trust in web platform–based campaigns to fund their medical expenses, but our review of the literature suggests that success is more likely linked to single cases, and is generally the consequence of a donation-based scheme or, at most, one that is reward-based, regardless of the health care system archetype (public, private insurance-based, or hybrid).

Equity crowdfunding is certainly disrupting the way many ventures, especially small to medium start-ups, seek capital on the market, thus proving itself to be a powerful instrument for the distribution of risk and the rewarding of socialization. At the same time, no relevant or consistent data are available on equity crowdfunding in health care, apart from a few anecdotal cases where platform- and crowd-raised funds have been used as a medium for seeding and angel investing in early biotech start-ups.

Our assessment of the literature indicates that many scholars and practitioners have been studying medical crowdfunding, exposing gaps in the economic and health care systems, especially those paid for by the public purse, arguing how far this on-trend practice can exacerbate and widen social inequalities. On the other hand, as the data suggest, crowdfunding is giving new hope to patients all over the world who are unable to access medical care, whilst helping small investors and donors to seed projects in the most unmet areas of medical need, such as rare diseases. We firmly believe that this may be the first step toward a different way of proceeding, a new ecosystem where stakeholders, from private and public investors to patients and society, play a new and different role in the sustainability of health care for the good of us all.

### Limitations and further developments

Over the past decades, investments in health care across all subsectors have been dramatically increasing. However, this vast appetite for funds has provided no assurance of a better outcome in public health or for the investors’ wealth. While economic and health care systems still struggle with accessible and affordable medical care, the industry’s returns on investment have been shrinking over time. Social responsibility, sustainable growth, fair access to medicines and the cost of medical care are still burning topics at the core of the global health care agenda. We believe that attempting to use change the approach to pricing and late-stage business models as the only hammer to crack health care funding will come up short of expectations.

With the caveat that our review only examined papers in English, and there may be studies in other languages that make different suggestions, the changes being proposed are merely tweaks and will not be sufficient on their own—at least not without a profound disruption in the way the health industry is currently funded and the way it engages with its stakeholders to act early on the many signals coming from evidence out in the real world.

The abundance of single-case, donation-based campaigns proves that the art of pricing is not enough to ensure fair access to treatment; added to which, putting up obstacles may threaten investment in life sciences and, ultimately, evolution in health care and, critically, in global health. Three main hypotheses were developed from our findings. The first hypothesis is that medical crowdfunding, specifically equity and lending crowdfunding, is still at an early stage and will naturally and gradually expand from small, isolated start-up applications to capital-heavy industries, involving health care in general and pharmaceuticals in particular, where R&D costs now make up over one billion dollars. The second hypothesis is that health care ventures are simply too capital intensive to be fruitful and sustainable to apply them to small-scale institutions, despite the fact that equity fundraising is already soaring in industries that are equally capital intensive, like real estate (Montgomery et al. 2018). The third hypothesis is that health care systems will leverage on the experience gained from Fintech/Regtech sandboxes, and will introduce similar schemes that try to remove regulatory uncertainty (Goo and Heo 2020) and enable entrepreneurship (Alaassar et al. 2020), thus, driving development while reducing the financing needs.

We thus recommend future research to develop along different directions. Firstly, empirical research on medical crowdfunding should be enriched. Secondly, we should analyse the determinants for success of each crowdfunding archetype, above all equity crowdfunding, at different levels (private and public investors, patients and society). Thirdly, the possibility of conceptualizing additional and innovative funding schemes that can draw on the wealth of experience acquired from medical crowdfunding should be investigated. Despite the limitations and gaps that need to be filled, this study may not produce an immediate result, but can at least open the eyes of those in power and possibly point them in the right direction.

## Footnotes

1 Although fundings are into the billions of euros, they far from cover the total cost for all compounds.

2 In broader terms, Fintech refers to the enabling technology (tech) for financial services (Fin). Fintech can be clustered into main business segments, such as money transfers, saving and investing, lending (Caselli and Negri 2021) and insurance, or according to the kind of technology, into big data, AI, biometrics and blockchain (Muganyi et al. 2022). Given the nature of this research, we are focusing here on access to finance through Fintech and, more specifically, on crowdfunding, as an alternative to traditional funding (Bouncken et al. 2015).
